# Proxies of CRISPR/Cas9 Activity To Aid in the Identification of Mutagenized Arabidopsis Plants

**DOI:** 10.1534/g3.120.401110

**Published:** 2020-04-14

**Authors:** Renyu Li, Charles Vavrik, Cristian H. Danna

**Affiliations:** * Department of Biology, University of Virginia, Charlottesville, Virginia, and; ^†^ Albemarle High School, Albemarle County, Virginia

**Keywords:** CRISPR mutants, selection, PROXY phenotypes, gene function, *Arabidopsis thaliana*

## Abstract

CRISPR/Cas9 has become the preferred gene-editing technology to obtain loss-of-function mutants in plants, and hence a valuable tool to study gene function. This is mainly due to the easy reprogramming of Cas9 specificity using customizable small non-coding RNAs, and to the possibility of editing several independent genes simultaneously. Despite these advances, the identification of CRISPR-edited plants remains time and resource-intensive. Here, based on the premise that one editing event in one locus is a good predictor of editing event/s in other locus/loci, we developed a CRISPR co-editing selection strategy that greatly facilitates the identification of CRISPR-mutagenized *Arabidopsis thaliana* plants. This strategy is based on targeting the gene/s of interest simultaneously with a proxy of CRISPR-Cas9-directed mutagenesis. The proxy is an endogenous gene whose loss-of-function produces an easy-to-detect visible phenotype that is unrelated to the expected phenotype of the gene/s under study. We tested this strategy via assessing the frequency of co-editing of three functionally unrelated proxy genes. We found that each proxy predicted the occurrence of mutations in each surrogate gene with efficiencies ranging from 68 to 100%. The selection strategy laid out here provides a framework to facilitate the identification of multiplex edited plants, thus aiding in the study of gene function when functional redundancy hinders the effort to define gene-function-phenotype links.

Gene function studies are essential to understand how the genetic makeup of an organism translates into traits. Because of its genetic tractability, assembled genome sequence and the availability of collections of mutants, the model plant *Arabidopsis thaliana* is the preferred plant species to uncover gene function. Arabidopsis is a diploid species with a genome size of ∼135 Mb and ∼29,000 genes that encode for ∼37,000 proteins. However, in spite of the three decades of effort by many research groups, less than 40% of the Arabidopsis genes have been experimentally linked to at least one phenotype (Li *et al.* 2012; Akiyama *et al.* 2014; Provart *et al.* 2016). Forward genetic screens of chemically (*i.e.*, EMS) or physically (*i.e.*, γ irradiation) mutagenized Arabidopsis have been very successful at connecting genes to protein function and phenotypes (Koornneef and Meinke 2010). However, reverse genetic screens of T-DNA insertional lines or transposon mutagenized lines have shown a more limited success, as most single gene mutants do not produce observable phenotypes (Lloyd and Meinke 2012). Although a diploid species, the Arabidopsis genome contains a large number of gene duplications (Arabidopsis Genome Initiative *et al.* 2000) that often translate into completely or partially redundant gene functions that need to be eliminated in the same genetic background in order to produce a phenotype (Arabidopsis Genome Initiative *et al.* 2000; Vision *et al.* 2000; Bowers *et al.* 2003).

Three gene targeting methods, Zinc Finger Nuclease (ZFN), Transcription Activator-Like Effector Nuclease (TALEN), and CRISPR/Cas9, have accelerated gene function discovery in the past few years. All three methods have been successfully used in Arabidopsis, and other plants, to generate loss-of-function (LOF) mutations in specific genes (Miller *et al.* 2007, 2011; Christian *et al.* 2010; Li *et al.* 2013). All three methods are based on directing the activity of a DNA endonuclease to a specific target sequence in the genome to generate a double strand break (DSB). This is followed by the repair of the DSB via Non-Homologous End-Joining (NHEJ) (Puchta 2005; Schiml and Puchta 2016). This error-prone DNA repair pathway generates indels that shift the frame of the coding sequence, which will likely eliminate the function of the encoded protein. While ZFN and TALEN DNA-binding specificity is based on DNA-binding protein domains that need to be optimized for every gene of interest, the gene specificity of CRISPR/Cas9 is provided by an easily customizable single guide RNA (sgRNA). Each sgRNA has two important elements: 1) a 20 bp gene-specific CRISPR RNA (crRNA) that defines the target sequence; 2) a constant *trans*-activating crRNA (tracrRNA) that provides with the secondary structure that mediates the formation of the DNA-sgRNA-Cas9 complex and the activation of Cas9 (Jinek *et al.* 2012; Karvelis *et al.* 2013). Hence, targeting a new gene with CRISPR/Cas9 only requires the identification of a 20mer gene-specific sequence located 3 bp upstream of a protospacer adjacent motif (PAM; a 5′-NGG-3′ for *Streptococcus pyogenes* Cas9) and the cloning of the crRNA upstream of the tracrRNA in the appropriate vector for plant transformation.

The expression of CRISPR/Cas9 in Arabidopsis and other plant species necessitates the insertion of foreign DNA encoding Cas9 and sgRNAs into the plant’s genome. For this, both Cas9 and sgRNAs are cloned in a binary vector and transgenic plants are obtained via *Agrobacterium tumefaciens*-mediated transformation of immature flowers (Clough and Bent 1998). In most vectors, Cas9 is expressed ubiquitously and constitutively by the Ubiquitin-10 (UBQ10) or the Cauliflower Mosaic-Virus 35S (CaMV-35S) promoters (Odell *et al.* 1985; Callis *et al.* 1995). Agrobacterium inserts the T-DNA randomly in the genome of a few cells, most of which are somatic cells. If some germline cells were transformed with the T-DNA, the transgene will pass to the next generation (T1 generation)(Hood *et al.* 1993). From the few plants in the progenies that receive the T-DNA, a yet smaller fraction would harbor the T-DNA in a region of the genome that allows for Cas9 expression. In the Cas9-expressing T1 plants, Cas9 will edit the target gene/s *in trans* in somatic cells as well as in some germline cells, thus allowing CRISPR-editing to pass to the next generation (T2 progenies). Finally, a small proportion of these T2 progenies will be homozygous for the mutation of interest. Usually, as the gene/s under study does not produce a known phenotype, the identification of the CRISPR-mutagenized T2 plants entirely relies on the non-biased DNA genotyping of a large number of plants (Thomas *et al.* 2014; Kim *et al.* 2014; Peng *et al.* 2018). A few alternatives have been pursued to alleviate the cost and to reduce the time invested in the identification of CRISPR-mutagenized plants. One such strategy is the use of Cas9 fusions to fluorescent proteins (*i.e.*, GFP, mCherry, etc.) or protein tags (*i.e.*, HA, FLAG, etc) to identify T1 plants that express Cas9 so that the expensive and time consuming molecular genotyping of plants can be focused on progenies of T1 plants that express Cas9 (Osakabe *et al.* 2016). These strategies inform Cas9 expression but do not alleviate the burden of screening large numbers of T2 plants, and typically requires imaging T1 plants under the microscope or detecting Cas9 protein fusions via western blot analysis (Osakabe *et al.* 2016). The direct PCR-amplification and DNA sequencing of target sequences in leaf tissue of T1 plants is often used as a strategy to marrow down the search to the progenies of T1 plants with confirmed edited targets. However, when Cas9 is transcribed from constitutive promoter, most T1 plants will be mosaics that do not pass the edited genes to the T2 generation (Feng *et al.* 2014, 2018). Therefore, the use of proxies of gene editing in the T2 progenies could significantly reduce the time and effort invested in identifying CRISPR-mutagenized plants.

CRISPR/Cas9 can simultaneously edit multiple loci (co-editing) at high frequency in the somatic tissues of T1 Arabidopsis and rice plants (Ma *et al.* 2015; Yan *et al.* 2016; Zhang *et al.* 2016; Minkenberg *et al.* 2017). Hence, we hypothesized that we could take advantage of this high co-editing frequency to aid in the selection of CRISPR-mutagenized plants. The rationale follows: if we targeted a gene that produces a visible and easy to detect phenotype, we could use it as a proxy to identify T2 plants where other loci of interest were simultaneously edited. To test this hypothesis, we chose three genes with independent functions and located in different chromosomes as potential proxies of CRISPR/Cas9 activity, namely *GLABRA-1* (*GL1*), *Jasmonic Acid Resistant-1* (*JAR1*) and *Ethylene Insensitive-2* (*EIN2*). In Arabidopsis, the formation of leaf trichomes is contingent to the function of *GL1*. LOF mutants of *GL1* do not produce trichomes, a phenotype that is easily observable as these plants have smooth leaves (Herman and Marks 1989; Marks and Feldmann 1989). LOF mutations in *JAR1* and *EIN2* produce insensitivity to the plant hormones jasmonic acid (JA) and ethylene (ET), respectively. The responses to both JA and ET can be monitored in seedlings exposed to JA or ET in tissue culture plates (Alonso *et al.* 1999; Staswick *et al.* 2002). In plants harboring a wild type allele of *JAR1*, exposure to JA causes root growth inhibition. In seedlings harboring a wild type allele of *EIN2*, exposure to ET causes hypocotyls to bend downward. We used these three genes to test whether they are effective proxies of CRISPR/Cas9 activity and could therefore facilitate the isolation of CRISPR-mutagenized T2 plants. Our data show that *gl1*, *ein2* and *jar1* proxies predict the editing of surrogate genes with frequencies ranging from 40–100%, greatly reducing the time and resources needed to identify CRISPR/Cas9 multiplex mutants for the genes of interest. Importantly, the selection strategy laid out in this study could accelerate the process and reduce the cost of identifying multiplex mutants in plant species with large and polyploid genomes.

## Material and Methods

### Design and synthesis of sgRNA expression cassettes

The sgRNAs were designed with an online web tool at the Zhang lab (crispr.mit.edu). The occurrence of the crRNA sequences retrieved by the software was verified via PCR amplification on genomic DNA from wild-type (Col-0) plants and Sanger sequencing of the PCR amplicon. Each candidate crRNA sequence was evaluated based on the calculated specificity score and the number of off-target sites (Table S1). Off-targets were predicted, based on sequence homology, as potential sites in the Arabidopsis genome where 5 based pairs out of the 20 nucleotides in the crRNA sequence could anneal to an unintended target. Bulge RNA was set to 0 to provide maximum stringency. The crRNA target site was inserted into an in-silico cloning construct template between the AtU6P promoter sequence and the tracrRNA sequence. The AtU6 promoter, crRNA, tracrRNA, and a poly-T tail together constitute a complete sgRNA expression cassette(Peterson *et al.* 2016). Each of the three individual cassettes was assembled into a stackable array. A 32-nucleotides sequence upstream of the first AtU6 promoter and a 17-nucleotide sequence downstream of the last poly-T tail of the array was included to facilitate future cloning (Figure S1). The final DNA sequence was synthesized through GenScript Custom Gene Synthesis services (Cat#SC1010).

### T-DNA construct and bacteria preparation

The synthetic sgRNA expression cassettes DNA fragment was PCR-amplified with high fidelity Taq DNA polymerase (NEB Phusion Cat#M0530S) using the forward and reverse primers 5′-aggctcccgggtgcgtcgacggtctcaggtcagagcttg-3′ and 5′-gaaagctgggtgattcaagcttggtctcatcagggatccaaaag-3′ respectively. The PCR fragment was assembled in a In-Fusion reaction (Takara In-Fusion HD Eco-Dry Cloning Plus Cat#638915) with a SalI linearized pDONR vector (NEB, SalI-HF Cat#R3138S) and HindIII (NEB, HindIII-HF Cat#R3104S) to obtain pDONR-CE, which contains the Gateway (GW) cloning sites attL1 and attL2. The sgRNA expression cassettes stacking was then inserted between the two GW cloning sites via In-Fusion reaction. Further, through GW LR recombination cloning (Thermo Fisher Scientific, Gateway LR Clonase Enzyme mix Cat#11791019), the entire sgRNA expression cassettes fragment was transferred into the binary vector (pCUT3), which encodes a maize optimized Cas9 enzyme linked to nuclear localization signal (NLS) and tagged to the HA epitope, under the control of a UBQ10 promoter [31].

### Plant transformation, selection and handling

All plants used in this study were Columbia-0 (Col-0) wild type background (Lehle Seeds, TX, Catalogue # WT-02). All transgenic plants were transformed with the pCUT3-CE binary vector via standard Agrobacterium-mediated floral dipping as previously described (Clough and Bent 1998). To select transgenic plants, T1 seeds were sown on sterile Petri dishes containing Murashige and Skoog (MS) medium 0.7% (w/v) phyto-agar (Plant Media Cat#40100072-2) and 50 µg/mL Kanamycin (Fisher Scientific CAS#25389-94-0). Seeds were surface sterilized with 10% (v/v) bleach and 0.1% Tween 20 (v/v). After 14 days of growth on sterile agar plate, resistant seedlings were scored and transferred to individual pots containing soil and were grown at 24°/16h light/100uM/cm2/sec^-1^ for further analysis and seed propagation. Green leaf tissue was collected 4 weeks later from each independent transgenic plant and stored at -80C for western blot analysis. T2 seeds were collected from individual T1 plants for further analysis. To visually select the glabrous plants (*gl1*), T2 seeds were stratified in 0.1% phytoagar at 4° for 3 days. Seeds were sown on commercial potting soil (50% Fafard + 50% MetroMIX 360, SunGro Horticulture) in 10” x 20” germination plastic trays without cells, using a 308-holes perforated basket as a matrix to evenly distribute seeds, which facilitates visual inspection after germination. Three trays (a total of 924 plants) were used per every T2 population screened. After 3 weeks at 24° / 16hr light photoperiod, glabrous plants were visually identified by the lack of leaf trichomes. For the visual identification of *jar1* and *ein2* T2 mutants, approximately 1000 seedlings per T2 progeny were screened in sterile petri dishes with MS medium supplemented with ACC (Millipore Sigma, SKU#A3903) or Methyl-Jasmonate (Millipore Sigma SKU#W341002) in tissue culture plates as previous described (Alonso *et al.* 1999; Staswick *et al.* 2002). Visually identified T2 seedlings were transferred to individual soil pots for further analysis and seeds propagation.

### Protein and western blot assay

Cas9 expression was assessed via Western blot in leaf samples. Total protein samples were extracted from 4-weeks old green leaf tissue via grinding in liquid nitrogen with mortar and pestle and resuspended in protein loading buffer (Tris-HCl, pH:8.8) and heated to 100° for 5min. Extracts were centrifuged at 17,000x g for 5min at 25° and the supernatant was used for gel blot analysis. Protein were subject to electrophoresis in poly-acrylamide (0.375M Tris-HCl pH = 8.8, 8% Acrylamide, 0.05% APS, 0.1% SDS) matrix and transferred to PVDF membranes (Thermo Scientific, Cat#88520) with 70V under 4° for 90min. After blocking in milk solution, the membranes were incubated with monoclonal rabbit anti-HA antibody (Cell Signaling Technology, mAb#3724, 1:4000 dilution) and monoclonal mouse anti-β-Actin antibody (Millipore Sigma, Cat#MAB1501). Secondary HRP conjugated anti-rabbit IgG antibody (Jackson Immuno Research Laboratories, Inc. Code#111-0350144) and dye conjugated (infrared) anti-mouse antibody (LiCor IRDye 800CW Goat anti-rabbit IgG P/N#925-32210) were used were used to detect Cas9-HA and beta-actin, respectively. ECL (Bio-Rad, Cat#1705060) chemiluminescence and infrared fluorescence were imaged with Bio-Rad ChemiDoc MP system and analyzed with Bio-Rad Image Lab software.

### Gene sequencing and allele detection

To detect CRISPR/Cas9 introduced mutations, gene specific primers annealing ∼300bp upstream and ∼300bp downstream the target site were used to PCR-amplify genomic DNA from visually selected plants with Phusion High-Fidelity DNA Polymerase (New England BioLab Inc., Phusion, Cat#M0530S). After the PCR, excess non-incorporated primers were removed with a single strand DNAase exonuclease (Fisher Scientific ExoSap-IT Cat#78-201-1ML), individual forward and reverse primers were added to separate Sanger sequencing reactions. Sanger sequencing was performed at Eurofin Genomic (Eurofins, Louisville, KY). The sequencing results (S1_Dataset) were analyzed using BLAST web tool at NCBI website, aligned against wild-type (Col-0) genomic sequence. All Sanger sequencing data are available at NCBI GenBank: nucleotide accession numbers MN411634 to MN412133. Fluorescence chromatograms were analyzed using the online tool ICE (Synthego, ICE Analysis; https://ice.synthego.com) to infer allelic composition of each T2 plant as described previously (Hsiau *et al.* 2019). Fluorescent chromatograms are available at NCBI Sequence Read Archive (SRA) accession number PRJNA575326.

### Analysis of sgRNA nucleotide composition and secondary structure

The calculation of G/C content of all sgRNAs that were used in the experiment were done by a Python script. The secondary structure was predicted by input the sgRNA FASTA sequence into the Mfold web server (http://unafold.rna.albany.edu) as previously described (Zuker 2003).

### Data availability

Supporting data, including Figure S1, Figure S2, Figure S3, Figure S4 and Table S1 are available through Figshare (10.6084/m9.figshare.12046920). Raw Sanger DNA sequencing data available at NCBI GenBank nucleotide accession numbers MN411634 to MN412133. Sanger DNA sequencing fluorescent chromatograms available at NCBI Sequence Read Archive (SRA) accession number PRJNA575326. Supplemental material available at figshare: https://doi.org/10.25387/g3.12030615.

## Results

### Design of an effective CRISPR/Cas9 co-editing proxy vector

**Figure 1:**
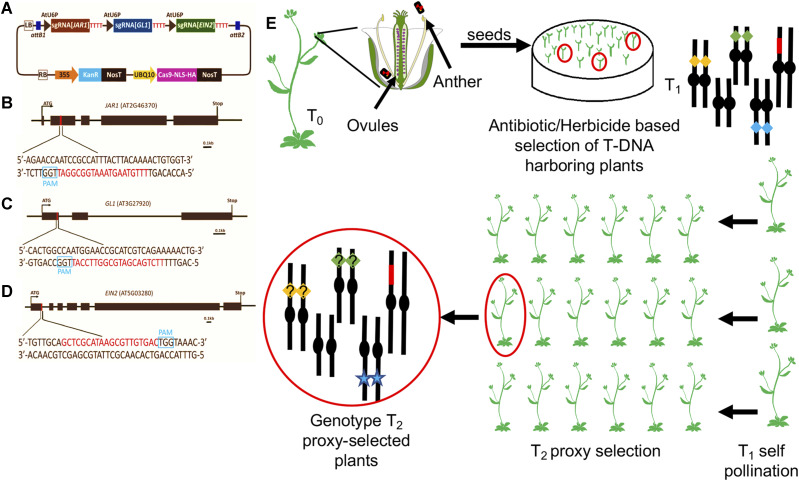
Proxy-based selection scheme of CRISPR-mutagenized plants. (A) The pCUT3-CE construct contains three individual transcriptional units that generate sgRNA for *JAR1*, *GL1* and *EIN2* editing. The expression of each sgRNA is controlled by individual AtU6 promoters (U6P) and poly-T terminator (TTTTTT). (B-D) *JAR1*, *GL1* and *EIN2* target sites. Sequence in red denote the 20nt crRNA target site within each proxy gene. The PAM site is boxed in blue. Scale bar = 0.1kb. (E) Selection scheme using proxy plants: T_0_ plants are transformed with the binary vector shown in (A). In the T1 generation, each gene targeted is depicted as a yellow, green or blue diamond, each one positioned on a different Arabidopsis chromosome. The T-DNA harboring CRISPR-Cas9 is depicted as a red chromosome fragment. The genotype of proxy-selected T2 plants is shown at the left end of the scheme: the blue star depicts loss-of-function mutation of the proxy gene that allowed for the visual selection of edited plants, while yellow and green diamonds with a question mark depict surrogate genes of unknow allelic condition that need to be PCR-genotyped.

A proxy-based CRISPR/Cas9 co-editing vector is composed of: 1) Cas9 nuclease coding sequence, 2) validated sgRNA against a proxy gene driven by the RNA polymerase-III promoter, 3) sgRNAs against all genes of interest, and 4) plasmid backbone encoding all elements required for Agrobacterium mediated transformation and selection of transformants (Clough and Bent 1998). To assemble each component of the multiplex gene targeting CRISPR/Cas9 system we used the all-in-one vector pCUT3-CE (Figure 1A)(Peterson *et al.* 2016). The resulting multiplex editing vector contains: 1) Cas9-HA fusion downstream of the UBQ10 promoter and upstream of NOS terminator; 2) three sgRNA expression cassettes within which each sgRNA is flanked by a U6 promoter (AtU6) and a transcriptional termination signal (“TTTTTT”) to provide similar expression levels across all three sgRNA. In addition, the RNA polymerase-III transcription start site “G” was added 23 nucleotides downstream of the AtU6 promoter TATA box to efficiently initiate the transcription of each sgRNA (S1 Fig); 3) all regulatory elements in the pCUT3 binary vector that allow for *E. coli* and Agrobacterium replication and provide Kanamycin resistance for the selection of transgenic T1 plants. To enable visual identification of CRISPR-mutagenized T2 plants we chose to mutagenize *GL1* (At3g27920), *JAR1* (At2g46370), and *EIN2* (At5g03280). Each of these loci reside in a different chromosome, their functions are unrelated to each other, and their LOF mutants can be visually identified. The 20nt crRNA sequences were designed to target either the 1^st^ or 2^nd^ exon of each gene to increase the likelihood of yielding a null allele as a consequence of NHEJ repair generating a frameshift or a premature stop codon for all potential isoforms of each gene (Figure 1B-D).

### Heritable CRISPR-Cas9-mutageniced proxies are easily detectable in T2 progenies

**Figure 2:**
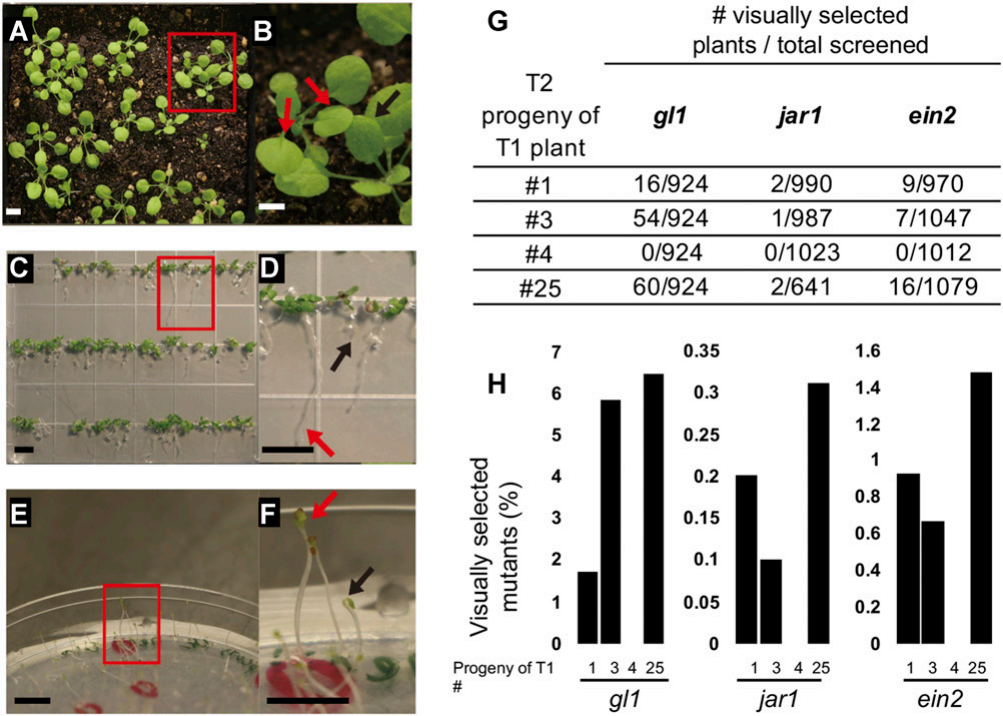
Visual identification of proxies of CRISPR-Cas9 mutagenesis. Proxy phenotypes of *gl1* (A and B), *jar1* (C and D) and *ein2* (E and F) loss-of-function mutants in T2 progenies. Red insets demark proxy edited plants side-by-side with wild type plants. Red insets closeups of *gl1*, *jar1* and *ein2* proxy plants are shown in (B), (D) and (F), respectively, with red or black arrows pointing toward mutant and wild type plants of each proxy, respectively. (G) Number of proxy plants identified in the T2 progeny of 4 independent T1 plants. (H) Percentage of loss-of-function mutants identified in T2 progenies of 4 independent T1 plants (#1, #3, #4, #25). For *gl1* screening, seeds were sown in 3 trays per T2 population using a 308-holes plastic matrix (924 plants in total). The total number of *gl1*, *jar1* and *ein2* plants recovered was 130, 5 and 32, respectively. Scale bars in A-F = 5 mm.

After Agrobacterium-mediated transformation of immature flowers of Col-0 wild type plants, we selected for transformants on phyto-agar plates using Kanamycin 50 µg/mL as the selective agent. From five independent transformation experiments, we recovered a total of twenty-six T1 transgenic plants. After self-fertilization, we randomly chose the progeny of four T1 lines (#1, #3, #4 and # 25) for the visual selection of CRISPR-edited T2 plants (Figure 2). For each of the four independent T1 lines, we grew ∼1000 T2 seeds to screen for LOF proxy mutants (Figure 2A-F). Among T2 plants visually inspected for lack of trichomes, *gl1* mutants appeared with frequencies of 0.017, 0.058 and 0.064 in the progeny of T1 lines #1, #3 and #25, respectively (Figure 2G). For *jar1* or *ein2* selection, we sown ∼1,000 T2 seeds on Murashige and Skoog phyto-agar plates (MS plates) containing either the JA-precursor Methyl Jasmonate (MeJA) or the ET-precursor Amino-Cyclopropane-Carboxylic acid (ACC), respectively. One week after germination on MeJA-containing square plates incubated vertically to allow for root length assessment, wild type seedlings produced roots of 0.5 ± 0.2 cm in length, while *jar1* positive control seedlings produced roots of more than 1.5 cm in length. T2 seedlings from individual T1 progenies with roots of more than 1.5 cm in length were selected for further analysis. Five days after sowing on ACC-containing plates incubated in the dark, wild type seedlings produced hypocotyls of approximately 0.5 cm in length with the typical downward cubature, while *ein2* positive control seedlings produced straight hypocotyls of more than 1 cm in length. T2 seedlings from individual T1 progenies with straight long hypocotyls were selected for further analysis. Again, we found *jar1* and *ein2* plants in the progeny of T1 lines #1, #3 and #25, but we did not identify mutants in the T2 progeny of T1 line #4 (Figure 2G-I). Western blots of leaf protein samples of each T1 line analyzed showed Cas9 expression in lines #1, #3 and #25, but no in T1 line #4, explaining the lack of observable phenotypes in the T2 progeny of this T1 line (Figure S2). As expected, each individual T1 plant expressed different levels of Cas9, a variation that may stem from the independent chromosomal location of the T-DNA in each T1 plant analyzed. Interestingly, the percentage of visually identified T2 plants varied across independent T1 lines but did not correlate with Cas9 expression. The frequency at which *jar1* and *ein2* plants appeared across T2 progenies of T1 plants #1, #3, and #25 ranged from 0.001 to 0.003 and 0.064 to 0.147 respectively (Figure 2G and H). Again, no mutants were detected across T2 progeny of T1 plant #4.

Due to the UBQ10 promoter activity, the constitutive and ubiquitous expression of Cas9 may produce edited cells or lineages in somatic and/or germline tissues, yielding mosaic T2 plants. To reduce the odds of genotyping mosaic edited plants, we only selected T2 plants that had no trichomes anywhere in the leaves or showed long roots (more than 1.5 cm) or long straight hypocotyl (Figure 2A and F). We reasoned that mutations in these T2 plants should have originated in T1 germline and hence their genetic makeup should be more uniform across reproductive and somatic tissues. To assess the genetic makeup of the selected mutants, plants were allowed to self-pollinate and produce T3 seeds for progeny tests. The T3 progenies of ten *gl1*, ten *ein2* and five *jar1* T2 plants were tested for *gl1*, *ein2* and *jar1* phenotypes, respectively. All tested T2 plants produced 100% mutant progenies (approx. 25-50 T3 seedlings per every T2 plant tested), which confirmed that the selected T2 plants were non-mosaic LOF mutants.

### Sub-optimal sgRNA secondary structure limits the efficiency of CRISPR/Cas9 editing

To understand the underlying causes that produced different mutagenesis frequencies across the three proxies, we investigated several factors known to have an impact in sgRNA efficiency. Although not experimentally tested, we reasoned that expression differences across the three sgRNA would not account for the observed differences in mutagenesis frequencies, because: a) each sgRNA is transcribed as an independent unit from identical U6 promoters and terminators; b) difference in sgRNA expression would likely stem from the relative position of each sgRNA expression unit within the T-DNA (5′-*JAR1-GL1-EIN2*-‘3), which did not correlate with the observed mutagenesis frequencies (*GL1*>*EIN2*>*JAR1*). Overall, it seems unlikely that differences in expression could account for the efficiency of each sgRNA. Three other factors, primarily affected by the gene specific crRNA sequence, are known to impact sgRNA efficiency: 1) number of crRNA off-targets; 2) crRNA GC content; 3) sgRNA secondary structure (Doench *et al.* 2016). The first factor under consideration, the specificity score, is reported as a whole number out of 100 perfect score. The three 20-nt crRNA sequences had similar values (cr*JAR1*: 98/100, cr*GL1*: 98/100, cr*EIN2*: 99/100). Notwithstanding the higher number of predicted off-targets for the *JAR1* crRNA, which could explain its low editing efficiency, the *GL1* crRNAs had more predicted off-targets than *EIN2* crRNAs (cr*JAR1*: 9, cr*GL1*: 5, cr*EIN2*: 3) and yet *GL1* mutagenesis frequency was sixfold higher than that of *EIN2*. In addition, assuming that there is competition for the same sgRNA across potential off-target sites, we would have expected *EIN2* to show the highest mutagenesis frequency, an expectation that was not supported by the evidence. Hence, the number of predicted off-targets *per se* does not seem to explain the mutagenesis frequencies of each proxy. Therefore, we should consider other factors. The G/C content of the three crRNAs varied substantially, being 35% for cr*JAR1*, 50% for cr*GL1*, and 55% for cr*EIN2*, suggesting that the low G/C content of cr*JAR1* could, at least in part, explain its low mutagenesis efficiency. However, the GC content for all 3 crRNAs falls within the optimal range of 30–80% (Liang *et al.* 2016), and the GC content of cr*GL1* and cr*EIN2* was only 5% different while the editing efficiency of *GL1* was up to sixfold higher than that of *EIN2*. Therefore, GC content does not seem to be a major determinant of the efficiency differences across the three sgRNAs. A third factor to consider is the secondary structure of the sgRNA. Previous studies revealed that three stem/loops (hairpins) are necessary for the formation of a DNA-sgRNA-Cas9 complex (Liang *et al.* 2016). Among these, stem loop #1 is crucial for the formation of a functional Cas9-sgRNA-DNA complex, while stem loop #2 is critical to improve complex stability and *in vivo* Cas9 activity. To analyze sgRNA secondary structure we performed *in-silico* analysis of the *JAR1*, *GL1* and *EIN2* sgRNAs sequences using the online tool Mfold (Zuker 2003). A single *in-silico* prediction revealed that all three stem loops are intact in the *GL1* sgRNA (Figure S3D). The *EIN2* sgRNA analysis rendered two alternative predictions (Figure S3E and F), one showing all three intact stem loops and one showing a missing stem loop #1. The secondary structure predictions of *JAR1* sgRNA showed that stem loops #1 and #2 are missing in all three alternative predicted structure (Figure S3A-C). Hence, the presence of three stem loops, concomitantly with the low number of consecutive base pairs (CBPs) and internal base pairs (IBPs) in *GL1* sgRNA may explain the higher efficiency of *GL1* mutagenesis compared to *EIN2* and *JAR1*. The presence of two stable and one unstable stem loop, together with the higher number of CBPs and IBPs, may explain the intermediate efficiency of the *EIN2* sgRNA. Finally, the absence of stem loops #1 and #2, and high number of CBPs and IBPs would explain the low efficiency of *JAR1* sgRNA (Figure S3A-C and Table S1). Therefore, as previously reported, stems/loops #1, #2 and #3, as well as a low number of CBPs and IBPs in the crRNA sequence, are likely critical features that must be considered at the time of choosing gene-specific crRNA sequences to design efficient sgRNAs (Liang *et al.* 2016).

### Efficient identification of Cas9 editing at independent loci using proxy-based selection

**Figure 3:**
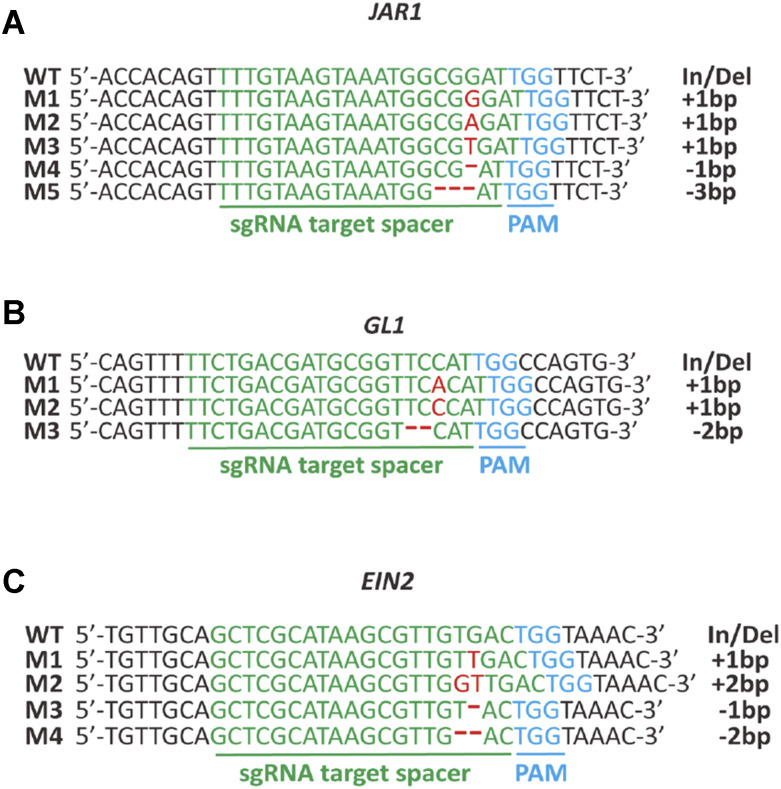
Sequence analysis of CRISPR/Cas9 edited proxy genes. CRISPR/Cas9 induced mutations detected via PCR and Sanger sequencing in *jar1* (A), *gl1* (B) and *ein2* (C) mutants across independent T2 progenies. The target sequence is depicted in red. The PAM site is depicted in blue. Indels are depicted in green. Wild type reference DNA sequence (WT) and mutant alleles (M1…5) detected for each proxy gene are shown as sequence alignments.

For most gene-editing endeavors, the phenotype(s) associated to the gene(s) of interest is not known. In fact, the purpose of using CRISPR/Cas9 editing in most cases is to uncover unknown phenotypes associated to the function of the gene(s) of interest. To verify the mutations at each locus, we analyzed the DNA sequences of visually selected *gl1*, *jar1*, and *eni2* plants. We collected leaf samples from 130 *gl1*, 32 *eni2* and 5 *jar1* T2 proxy plants, extracted DNA, and PCR-amplified 600bp of DNA surrounding the target sequence of the three genes. A CRISPR-edited target typically consists of a mix of mutant and wild type sequences (alleles). This complexity stems from DSB of DNA introduced by Cas9 followed by DNA repair via NHEJ, a process that may start when the T-DNA harboring CRISPR/Cas9 is integrated into the genome of germline cells in T0 plants. Throughout the T1 and T2 generations, Cas9 will continue to edit wild type target sequences in germline as well as somatic cells. The analysis of the DNA sequences was accomplished using the ICE (Inference of CRISPR Edits) sequence analysis tool (https://ice.synthego.com). ICE weighs the quality of each DNA sequence and determines the relative representation of each of the four possible nucleotides at each given position in the DNA sequence via assessing the area below the fluorescence chromatogram peak at each position (Figure S4)(Hsiau *et al.* 2019). Among the visually selected *gl1*, *jar1* and *eni2* plants, we identified three different alleles of *gl1*, five alleles of *jar1*, and four alleles of *eni2* (Figure 3). The mutations detected in the three proxies were consistent with previous studies reporting insertions of 1 or 2 nucleotides (+1 or +2) and deletions of 1, 2 or 3 nucleotides (-1, -2 and -3) within the six nucleotides upstream of the PAM sites, which are the hallmarks of NHEJ-mediated DNA repair in Arabidopsis (Jinek *et al.* 2012; van der Oost 2013; Peterson *et al.* 2016).

To assess the power of each of these proxies to predict co-editing in surrogate genes, we PCR-amplified and sequenced surrogate genes in every proxy selected plant (167 proxy plants). Sequences were analyzed using ICE CRISPR analysis software to detect edited alleles in surrogate genes. We PCR-amplified and sequenced: a) *JAR1* and *EIN2* target sequences in 130 *gl1* proxy plants; b) *JAR1* and *GL1* target sequences in 32 *ein2* plants; c) *EIN2* and *GL1* targets in 5 *jar1* proxy plants. We determined the frequency of double and triple editing (co-editing) by counting the number of proxy plants with edited sequences upstream of the PAM site of each surrogate target. A detailed analysis of the DNA sequence of each surrogate gene revealed a complex allelic composition. As expected, we detected non-edited wild type (wt) alleles, edited alleles in homozygosity (HM) and edited alleles in heterozygosity (het), including bi-allelic or higher order allelism (Bi) for each surrogate gene across different proxy plants. Most proxy plants had at least one edited surrogate gene (Table 1). Among *gl1* proxy T2 plants, 79% had *EIN2* edited alleles and 35% had *JAR1* edited alleles. Importantly, 15% of the *gl1* plants were *gl1-ein2* double HM. Among *ein2* proxy plants, 34% had *GL1* edited alleles and 25% were *ein2-gl1* double HM. Of the 5 *jar1* proxy plants recovered, all had *GL1* and *EIN2* edited alleles, but no double or triple HM were identified. Triple edited plants were also found among most proxy plants, with percentages of 79% (103/130), 100% (5/5) and 31% (10/32) for *gl1*, *jar1* and *ein2* proxy plants, respectively (Table 2). Most impressive, 1 of the 102 triple-edited *gl1* plants was a triple HM (no *EIN2* or *JAR1* wt alleles were detected). Among *ein2* proxy plants, 12% (4/32 plants) were double HM edited plants (did not have *JAR1* or *GL1* wt alleles). Although JA-insensitive plants (*jar1*) were recovered from every of the three independent T1 progenies (#1, #3 and #25), we only recovered a total of 5 *jar1* plants (Figure 2G). Notwithstanding the low number, every *jar1* proxy plant recovered also carried *GL1* and *EIN2* mutant alleles in heterozygosity, suggesting a high predictive power for this proxy (Table 1 and Table 2).

**Table 1:** Double-editing scoring in proxy selected plants

		Alleles Detected*^a^*					Alleles Detected*^a^*			
Proxy Selection	Surrogate Gene	wt*^b^*	HM*^c^*	Het*^d^*	Bi*^e^*	Surrogate Gene	wt*^b^*	HM*^c^*	Het*^d^*	Bi*^e^*
*gl1* (130)*^a^*	*EIN2*	28	20	79	3	*JAR1*	84	1	44	1
*jar1* (5)*^a^*	*GL1*	0	0	5	0	*EIN2*	0	0	5	0
*ein2* (32)*^a^*	*GL1*	11	8	13	0	*JAR1*	11	8	13	0

a Number of *gl1*, *jar1* and *ein2* proxy selected plants bearing wild type or edited alleles of surrogate genes as indicated.

b Only wild type alleles detected.

c No wild type allele detected.

d Both wild type and edited alleles detected.

e Two different edited alleles detected / No wild type allele detected.

**Table 2:** Triple-editing scoring in proxy selected plants

Proxy Selection	Surrogate Genes	wt/wt*^b^*	HM/HM*^c^*	HM/Het*^d^*	HM/Bi*^e^*	HM/wt*^f^*	Het/Het*^g^*	Het/Bi*^h^*	Het/wt*^i^*	Bi/Bi*^j^*	Bi/wt*^k^*
*gl1* (130)^*a*^	*EIN2* and *JAR1*	27	1	10	0	9	30	2	49	0	2
*jar1* (5)^*a*^	*GL1* and *EIN2*	0	0	0	0	0	5	0	0	0	0
*ein2* (32)^*a*^	*GL1* and *JAR1*	10	0	4	0	4	6	0	8	0	0

a Number of *gl1*, *jar1* and *ein2* proxy selected plants bearing wild type or edited alleles of surrogate genes as indicated.

b Only wild type alleles detected for both surrogate genes.

c No wild type alleles detected for any of the two surrogate genes.

d No wild type alleles detected for one surrogate gene / both wild type and edited alleles detected for a second surrogate gene.

e No wild type alleles detected for one surrogate gene / two or more different edited alleles for the second surrogate gene.

f No wild type alleles detected for one surrogate gene / only wild type alleles detected for the second surrogate gene.

g Both wild type and edited alleles detected for both surrogate gene.

h Both wild type and edited alleles detected for one surrogate gene / two or more different edited alleles for the second surrogate gene.

i Both wild type and edited alleles detected for one surrogate gene / only wild type alleles detected for the second surrogate gene.

j Two or more different edited alleles detected for both surrogate genes.

k Two or more different edited alleles detected for one surrogate genes / only wild type alleles detected for the second surrogate genes.

The data presented in Tables 1 and 2 allows us to hypothesize that focusing the DNA genotyping on proxy-selected plants, greatly increases the odds of finding multiplex edited plants as opposed to randomly genotyping T2 plants. To test this hypothesis, we calculated the expected frequency at which double and triple LOF mutants would appear in the T2 population and compared that frequency with the observed frequency at which they appeared in proxy-selected plants. In the absence of co-editing, that is, in a case where each gene is target independently in different cells of the germ line, the proportion of double and triple mutants in T2 plants will be the mathematical product of the proportion at which each single mutant appears in the T2 generation. In our study, *gl1*, *jar1* and *ein2* LOF mutants appeared at frequencies of 0.04, 0.002 and 0.01, respectively (Figure 2G). Hence, the expected frequencies of *gl1-ein2*, *gl1-jar1* and *ein2-jar1* double mutants are 0.00047, 0.000092 and 0.000019, respectively. However, the observed frequencies at which these mutants appear among proxy plants was 0.16, 0.37 and 0.018, respectively, between 2 and 4 orders of magnitude higher than the expected frequencies (Table 3). For triple LOF mutants, while the expected frequency would be 8.96E-07, the observed frequency among proxy-selected plants was 0.004, four orders of magnitude higher than expected (Table 3). To test if observed and expected values were statistically similar (null hypothesis) we run a “Hypothesis Test of one Proportion” analysis. The null hypothesis (similarity) for expected *vs.* observed proportions for each double LOF mutant (*gl1-ein2*, *gl1-jar1*, *jar1-ein2*) and that of the triple LOF mutant (*gl1-ein2-jar1*) was falsified in every case with a “p value” smaller than 0.0001 (Table 3). These data strongly support the claim that focusing the DNA genotyping analysis on proxy selected plants, as opposed to randomly chosen T2 plants, significantly increases the odds of finding edited alleles in surrogate genes (genes of interest). Overall, our data demonstrate that proxies of CRISPR mutagenesis are powerful predictors of the occurrence of CRISPR-induced editing in surrogate genes, thus allowing for the rapid identification of plants where genes of interest have been edited.

**Table 3:** Predictive co-editing power of proxy plants

Double edited						Triple edited	
*gl1;ein2*		*gl1;jar1*		*ein2;jar1*		*gl1;ein2;jar1*	
Exp*^a^*	Obs*^b^*	Exp*^a^*	Obs*^b^*	Exp*^a^*	Obs*^b^*	Exp*^a^*	Obs*^b^*
4.479E-04	0.1675(✻)	9.27E-05	0.0107(✻)	1.93E-05	0.0185(✻)	8.96E-07	0.0042(✻)

a Expected frequencies at which double and triple edited plants, in this case LOF mutants, would appear in the T2 population calculated as the product of the frequency at which each single mutant appeared in the T2 plants.

b Observed frequencies at which double and triple edited plants, in this case LOF mutants, actually appeared in T2 proxy selected plants.

^✻^ Z-test statistically significant at *P* ≤ 0.0001 with a 95% confidence interval.

## Discussion

Since first introduced as a new technology to produce LOF mutants, it was recognized that the identification of CRISPR-edited plants was time consuming and onerous. To alleviate this burden, previous studies have focused on developing Cas9-protein fusions to identify plants that express Cas9 (Osakabe *et al.* 2016; Tang *et al.* 2018). However, these strategies only inform of Cas9 expression rather than editing activity and involve imaging leaf samples with a fluorescence microscope or taking leaf samples to perform western blots. In addition, a large number of T2 progenies of the Cas9 expressing plants need to be DNA-profiled in order to identify the T2 plants harboring mutant alleles. Our proxy-based selection strategy greatly reduces the number of plants that need to be DNA profiled: the analysis of 1 in 2 visually selected plants would be enough to identify mutations in a second or a third gene of interest. Previous studies reported high co-editing frequencies when two or more genes were targeted with sgRNAs expressed from the same construct (Feng *et al.* 2014; Zhang *et al.* 2016). Indeed, the frequency of double and triple edited genes detected across proxy plants (Table 1) was several orders of magnitude higher than that expected in the absence of co-editing, demonstrating the advantage of focusing the DNA genotyping to proxy plants instead of blindly screening for edited plants (Table 3). A large CRISPR mutagenesis study that targeted a *GL1* homolog (*GL2*) in Arabidopsis has used PCR sequencing to screen 968 plants obtained from 10 independent T2 progenies of pre-selected T1 plants (approx. 100 plants per progeny). Only 55 T2 plants had *gl2* edited alleles and all of them were chimeric plants (Feng *et al.* 2018). These data clearly show that, even focusing the screening of T2 plants on progenies of pre-selected T1 plants, it is extremely unlikely to randomly pick T2 plants that have been edited in one gene, much more so in two or three genes of interest. With our proxy-based selection, every proxy plant recovered had mutations in at least one, and the majority of them had mutations in two surrogate gene (Table 1 and Table 2).

A major improvement in editing frequencies has been achieved by expressing Cas9 at high levels in specific cells that will pass the genome editing to the next generation of plants. The high and cell-specific expression of Cas9 driven by egg cells specific promoters have greatly increased CRISPR editing frequency, thus reducing the burden of identifying edited plants (Xing *et al.* 2014). In addition, the Ribosomal Protein S5-A (RPS5A) promoter has also been used to constitutively drive the expression of Cas9 producing high editing frequencies (Tsutsui and Higashiyama 2017). The high expression of Cas9 provided by RPS5A however, may lead to an increase in off-target editing. Indeed, it has been recently uncovered that the high expression of Cas9 driven by egg cell specific promoters produces an unusually high number of off-target editing events, an undesired effect never reported previously for constitutive promoter-driven Cas9 expression (Zhang *et al.* 2018). Therefore, it seems likely that constitutive promoters that express low levels of Cas9 will remain the preferred option for CRISPR/Cas9 mutagenesis. In anticipation to the potentially high interest in the proxy-based selection strategy described here, we have made and tested a binary vector that expresses Cas9 under the control of the UBQ10 promoter and an sgRNA that targets *GL1*. Potential users will be able to clone sgRNAs to target their genes of interest via Gateway in the same vector that targets *GL1*.

As for the most effective proxy-based selection strategy, some considerations may be necessary. For instance, the most effective sgRNA was the one targeting *GL1*. However, when looking at the frequency of co-editing, although high (∼80% of the *gl1* plants had mutations in a second gene, and ∼40% had mutations in a second and a third gene of interest), *gl1* was less effective than *jar1* at predicting editing events in surrogate genes. All five *jar1* plants isolated in this study had mutations in both *GL1* and *EIN2*. This is likely due to the low mutagenesis observed in *JAR1* combined with the higher mutagenesis observed in *GL1* and *EIN2*. Although not experimentally tested in this study, our analysis suggests that the mutagenesis frequency of each gene tested correlates with its sgRNA secondary structure, which in turns is highly dependent on the gene-specific crRNA sequence. In light of this observation, it seems reasonable to hypothesize that using a suboptimal (low efficiency) sgRNA to target the proxy of choice, in combination with highly efficient sgRNA/s to target the gene/s of interest, could dramatically reduce the number of plants that need to be DNA profiled to identify CRISPR multiplex mutants. Our results, in combination with previous reports showing that several genes can be Cas9 mutagenized simultaneously, support the idea that proxy-based selection could be scaled up to target more than three genes at a time (Feng *et al.* 2014; Zhang *et al.* 2016).

Unlike Cas9 fusions to fluorescent proteins or epitope tags, the identification of *jar1*, *gl1* and *ein2* mutant plants does not require any sophisticated piece of equipment. While *jar1* and *ein2* plants can be identified in culture plates within 1 week after germination, the identification of *gl1* mutants only involves growing T2 plants in soil for three weeks and visually identifying plants with smooth leaves among hairy wild type plants, thus, making this strategy more accessible for laboratories with few resources or even the school classroom. The choice of the appropriate proxy will depend on the plant species and on the phenotypes expected/predicted to be associated to the gene/s of interest. The physical proximity of the proxy and the gene(s) of interest will facilitate the identification of CRISPR mutants as both, the gene of interest and the proxy will segregate together, providing maximum proxy prediction power. In this study, the proxies of choice are all located in different chromosomes to avoid co-segregation, which would have resulted in an artificially high predictive power for each proxy. Choosing a biologically neutral proxy is more critical than choosing a proxy that co-segregates with the gene(s) of interest. In this study, *JAR1* and *EIN2* were only chosen because of the easily selectable phenotypes of their LOF mutants, which was a necessary condition to test our hypothesis. However, both *JAR1* and *EIN2* encode key proteins in the JA and ET signaling pathways that integrate multiple responses to biotic and abiotic stress, and hence, they could affect the function of the genes of interest and/or contribute with undesirable phenotypes. On the contrary, *GL1* could be the proxy of choice in plant species where this gene function is needed for leaf trichomes development. LOF mutant alleles of *GL1* are found in many naturally occurring accessions of Arabidopsis, which suggests that *gl1* mutations could be left in the mutant background without causing detrimental or pleiotropic effects that could interfere with future gene function studies, unless of course, the genes of interest also affect trichome development. In any event, the proxy mutation could be segregated from the genes of interest via backcrossing the CRISPR mutagenized line with wild type plants. The entire process of cleaning the background of the proxy mutation will be further slowed down if the proxy and the genes of interest were linked in the same chromosome arm. Overall, cleaning the genetic background to remove the proxy mutation would defeat the purpose of using proxy-based selection. A valid alternative would be the use of a proxy with no biological function (*i.e.*, a fluorescent protein). Transgenic seeds expressing the red fluorescent proteins mCherry or dsRED can be easily identified using an inexpensive green LED flashlight and a red filter (Lu and Kang 2008; Gao *et al.* 2016). LOF mutants for such proxy could be visually identified in T1 or T2 seeds of CRISPR mutagenized plants as fluorescence-less seeds. As the fluorescent protein works itself as a genetic transformation marker, the transgenic background expressing mCherry or dsRED would not need to harbor any antibiotic or herbicide resistance gene, thus facilitating the use of antibiotic or herbicide resistance markers for future genetic transformation with a binary vector harboring Cas9 and the sgRNAs.

The strategy laid out in this study constitutes a proof-of-principle of the potential use of proxies to facilitate the selection of genome-edited plants. Similar selection strategies could be used for the identification of CRISPR/Cas9 mutagenized plants in crops with more complex, often polyploid genomes, where the identification of multiplex mutants remains challenging.
